# Sugarcane vinasse remediation through HA–nCaO within a computational sustainability and green SDG framework

**DOI:** 10.1038/s41598-025-26216-5

**Published:** 2025-11-27

**Authors:** Mahmoud F. Mubarak, Ahmed M. Saleh

**Affiliations:** 1 Petroleum Applications Department, Egyptian Petroleum Research Institute (EPRI), Ahmed El-Zomer, Nasr City, Cairo Egypt; 2 Pharmaceutical Chemistry Department, Faculty of Pharmacy, Horus University, Horus, 34518 Egypt

**Keywords:** Vinasse treatment, Humic acid, Nano-calcium oxide, COD reduction, Organic pollutant removal, UN sustainable development goals, Need quality sustainability (NQS) index., Chemistry, Engineering, Environmental sciences, Materials science

## Abstract

The disposal of sugarcane vinasse, a highly acidic distillery effluent, presents a serious environmental management challenge. In this study, a novel composite adsorbent was developed by integrating green-synthesized nano-calcium oxide (nCaO) with humic acid (HA) via an aqueous co-precipitation method and ultrasonic-assisted stabilization. Characterization (XRD, FTIR, SEM, BET) confirmed the formation of a mesoporous hybrid with a surface area of 112.6 m² g⁻¹, average pore size of 6.3 nm, and enhanced surface basicity due to HA functionalization. Batch adsorption experiments demonstrated that an optimal dosage of 5 g L⁻¹ achieved 82.4% COD removal, 76.1% TOC reduction, and 89.5% color removal within 90 min, surpassing conventional treatments such as lime neutralization, Fenton oxidation, anaerobic digestion, and biochar systems under comparable conditions. The acidic vinasse (pH < 4) was neutralized to 7.6 ± 0.2 without the addition of external alkali. The incorporation of a chalcogel stage further promoted precipitation of suspended solids and volatile fatty acids, reducing residual organic load. Kinetic modeling indicated a pseudo-second-order fit (R² = 0.991), suggesting a chemisorption-dominated mechanism coupled with acid–base neutralization and partial precipitation. Reusability testing confirmed ≥ 68% efficiency retention after four cycles. Sustainability evaluation using the Need Quality Sustainability (NQS) index and Koel’s Pyramid metrics indicated favorable environmental and operational performance, aligning with UN SDG 6 (Clean Water and Sanitation) and SDG 12 (Responsible Consumption and Production).

**Supplementary Information:**

The online version contains supplementary material available at 10.1038/s41598-025-26216-5.

## Introduction

The rapid growth of bioethanol production, especially from the sugarcane and sugar beet sectors, has led to the accumulation of large volumes of vinasse, a dark, acidic, and highly concentrated organic by-product^1^. For each liter of ethanol produced, around 8 to 15 L of vinasse are produced. This fluency is marked by an extremely high chemical oxygen demand (COD > 30,000 mg/L), a low pH range (3.5 to 5.0), elevated total solids, significant color, and high concentrations of recalcitrant compounds, including polyphenols, melanoidins, and volatile fatty acids^2–4^. Inadequate treatment or indiscriminate discharge of vinasse can lead to severe threats to aquatic ecosystems and agricultural lands, primarily due to its phytotoxicity, high organic load, and potential for groundwater contamination^5,6^. Traditional treatment methods such as anaerobic digestion, and advanced oxidation processes (AOPs) have been applied to vinasse remediation. Nonetheless, these techniques are frequently hindered by limitations such as high operational costs, extended retention times, the production of secondary pollutants, and incomplete mineralization of organic compounds^7^. Recently, nanotechnology has gained attention as a promising alternative for wastewater treatment, providing high surface reactivity, improved adsorption capacity, and the ability to target specific contaminants effectively^8–12^.Among the various nanomaterials, nano-calcium oxide (nCaO) has garnered significant interest due to its strong basicity, broad availability, low toxicity, and multifunctional properties as an adsorbent, coagulant, and pH stabilizer^13–16^. However, the direct application of nCaO in aqueous systems can be challenged by issues such as particle agglomeration, limited colloidal stability, and inadequate selectivity for complex organic matrices like vinasse. Vinasse has been treated using traditional methods like anaerobic digestion and AOPs, but challenges remain due to its harsh physicochemical properties that harm the environment. Nano-calcium oxide-based adsorbents have shown promising results^17^. The HA–nCaO composite, with active groups such as carboxyl, hydroxyl, and phenol, enhances adsorption through chemisorption, offering an efficient and eco-friendly alternative^18^. To overcome these limitations, researchers have explored the use of organic modifiers and stabilizers. Humic acid (HA), a naturally occurring and environmentally friendly macromolecule derived from decomposed organic matter, has shown significant potential in stabilizing nanoparticles. This is attributed to its high molecular weight, presence of carboxylic and phenolic functional groups, and strong metal-organic complexation capabilities^19^. Notably, HA also exhibits effective adsorptive properties toward both organic and inorganic pollutants^20^. Despite the individual benefits of nCaO and HA in water treatment, a notable research gap exists in the development and application of HA-modified nano-CaO composites specifically for the targeted treatment of sugarcane vinasse.

To the best of our knowledge, no comprehensive study has yet reported the synergistic integration of humic acid and nano-calcium oxide into a hybrid colloidal composite designed specifically for the remediation of vinasse. This study introduces, for the first time, a novel composite designed for the sustainable treatment of sugarcane vinasse. The composite shows high performance in removing COD and TOC, decolorizing the effluent, and neutralizing its acidic pH without relying on conventional alkaline additives. It incorporates chalcogel to reduce the organic load through a chemisorption process and demonstrates strong reusability over multiple regeneration cycles. The research focuses on synthesizing an eco-friendly humic acid–modified nano-calcium oxide (HA–nCaO) composite and evaluating its efficiency in pH adjustment, COD reduction, and organic matter stabilization in vinasse treatment. The specific objectives of this study are as follows: First, to synthesize and characterize the HA–nCaO composite using techniques such as XRD, FTIR, SEM, and BET to determine its structural, morphological, and surface properties; Second, to evaluate the performance of the composite in batch experiments under varying conditions, including dosage, time, pH, and temperature; Third, to assess the removal efficiency of key vinasse pollutants, such as COD, TOC, color, and acidity; Fourth, to model the adsorption and reaction kinetics to better understand the mechanisms governing pollutant removal; and Fifth, to investigate the reusability and stability of the composite over multiple treatment cycles for potential large-scale applications. In accordance, for the first time, a tri-axial sustainability evaluation using the newly launched advanced software metrics was applied to offer a comprehensive comparison between the proposed approach and conventional hybrid beads, allowing for an innovative axis in-depth analysis across multiple dimensions for sustainability with computational evaluation^21–26^. This study presents a pioneering comparison that incorporates the United Nations Sustainable Development Goals (SDGs) alongside calculations of Koel’s pyramid axis, using the newly launched Need, Quality, and Sustainability (NQS) index. Each methodology is benchmarked for the greenness, blueness, and whiteness criteria, providing a comprehensive evaluation that integrates sustainability and practical relevance for unified sustainable applications^27,28^. Through this integrated approach, the study presents a cost-effective, environmentally friendly, and sustainable nanotechnology-based solution for addressing the environmental challenges posed by vinasse discharge in bioethanol production industries.

## Experimental

### Materials

All chemicals and reagents employed in this study were of analytical grade. Calcium oxide (CaO) nanoparticles, with a purity of ≥ 99.5% and particle size below 50 nm, were procured from Sigma-Aldrich (USA). Humic acid (HA), sourced from leonardite and containing ≥ 90% total humic substances, was obtained from Humintech GmbH (Germany). Ethanol (≥ 99.9%, HPLC grade) and sodium hydroxide pellets (≥ 98%) were supplied by Loba Chemie Pvt. Ltd. (India). Deionized water with a resistivity of 18.2 MΩ·cm was used in all synthesis and treatment processes. Vinasse employed in this study was sourced from the Egyptian Sugar and Integrated Industries Company (ESIIC), a prominent producer of sugar and ethanol within the Middle East and North Africa (MENA) region. The effluent was sampled directly from the terminal outlet of the distillation process during peak operational periods to ensure minimal variability in composition. To eliminate large particulate matter, the vinasse was passed through Whatman No. 1 filter paper. The clarified liquid was then preserved at 4 °C in sealed polyethylene containers to prevent chemical alteration prior to use. All glassware employed in the experimental procedures was meticulously cleaned using 1 M nitric acid (HNO₃) followed by repeated rinsing with deionized water to eliminate potential contaminants. The synthesis of the composite material, along with all batch experiments, was carried out under strictly controlled laboratory conditions to ensure experimental reproducibility and the reliability of the obtained data.

### Synthesis of HA–nCaO composite

The humic acid-modified nano-calcium oxide (HA–nCaO) composite was synthesized through a two-step procedure, which included a precipitation-dispersion step followed by functionalization via ultrasonication. Initially, 1.0 g of high-purity CaO nanoparticles was suspended in 100 mL of deionized water and subjected to vigorous magnetic stirring at 500 rpm for 30 min at room temperature (25 ± 2 °C) to achieve uniform dispersion. Simultaneously, a humic acid solution was prepared by dissolving 0.2 g of humic acid in 50 mL of deionized water, with continuous stirring and gentle heating (40 °C), until complete dissolution was attained. The HA solution was subsequently added dropwise to the CaO suspension under continuous stirring, promoting surface adsorption and facilitating the complexation between Ca²⁺ ions and the functional groups of HA (–COOH and –OH). Upon complete addition, the resultant brownish suspension was subjected to ultrasonication (Ultrasonic Bath, 40 kHz, 150 W) for 60 min to intensify nanoscale interactions, mitigate agglomeration, and facilitate the formation of a homogeneous organic-inorganic hybrid structure. The suspension was subsequently aged at ambient temperature for 12 h to ensure complete surface functionalization and stabilization. The composite was then isolated via centrifugation at 5000 rpm for 10 min, followed by three washing cycles with deionized water to eliminate any unbound humic acid. The material was then subjected to drying in a vacuum oven at 60 °C for a duration of 24 h. The dried composite was carefully ground in an agate mortar and subsequently stored in airtight containers for subsequent characterization and application in vinasse treatment experiments.

### Characterization techniques

The synthesized HA–nCaO composite was systematically analyzed using a range of physicochemical and morphological techniques to verify its structural characteristics, surface functionalities, and dispersion uniformity. X-ray diffraction (XRD) analysis was conducted utilizing a PANalytical X’Pert PRO diffractometer (Netherlands), employing Cu-Kα radiation (λ = 1.5406 Å) under operating conditions of 40 kV and 30 mA. This technique was used to identify the crystalline phases present and to verify the structural integrity of CaO following its modification with humic acid. Fourier-transform infrared spectroscopy (FTIR) analysis was performed using a Bruker Tensor II spectrometer (Germany) within the wavenumber range of 400–4000 cm⁻¹. This technique was employed to examine the surface functional groups and to confirm the interaction between CaO and humic acid, as evidenced by the presence of characteristic absorption peaks. Scanning electron microscopy (SEM) combined with energy-dispersive X-ray spectroscopy (EDS) was performed using a JEOL JSM-6510LV (Japan) to analyze the surface morphology, particle size distribution, and elemental composition of the composite. Brunauer–Emmett–Teller (BET) analysis was performed using a Micromeritics ASAP 2020 analyzer (USA) to evaluate the specific surface area, pore volume, and pore size distribution of the composite. Nitrogen adsorption–desorption isotherms were obtained at 77 K. Additionally; zeta potential measurements were conducted using a Malvern Zetasizer Nano ZS (UK) to assess the colloidal stability and surface charge of the composite in aqueous media. Thermogravimetric analysis (TGA) was performed using a Shimadzu DTG-60 H analyzer (Japan) under a nitrogen atmosphere, with a temperature range of 25 to 800 °C and a heating rate of 10 °C/min. This analysis was carried out to assess the thermal stability and organic content of the composite. The results of these comprehensive analyses collectively confirmed the successful synthesis of a stable, porous, and functionalized hybrid nanomaterial, demonstrating its suitability for high-efficiency organic pollutant removal from vinasse.

### Batch treatment procedure

Batch experiments were performed to assess the effectiveness of the synthesized HA–nCaO composite in treating raw sugarcane vinasse. The experiments were conducted in 250 mL Erlenmeyer flasks, each containing 100 mL of pre-filtered vinasse and varying concentrations of the HA–nCaO composite (ranging from 1.0 to 10.0 g/L). The flasks were agitated on an orbital shaker (IKA KS 4000 i control, Germany) at 150 rpm, maintained at a controlled ambient temperature of 25 ± 1 °C. The initial pH of the vinasse, typically around 4.2 ± 0.1, was recorded before treatment, with no external pH adjustments made to assess the inherent neutralization capacity of the composite. In the optimization studies, key parameters including contact time (ranging from 15 to 180 min), composite dosage, and initial pH were systematically varied to determine their influence on the performance of the HA–nCaO composite in treating raw sugarcane vinasse. At predetermined time intervals, 5 mL aliquots were withdrawn from each flask, filtered through 0.45 μm membrane filters, and subsequently analyzed for key water quality parameters. The Chemical Oxygen Demand (COD) was determined using the closed reflux colorimetric method, following the procedures outlined in Standard Methods (APHA, 2017). Total Organic Carbon (TOC) was quantified using a Shimadzu TOC-L analyzer, while color removal was evaluated by measuring the absorbance at 475 nm using a UV-Vis spectrophotometer (Jenway 7305, UK). The final pH values were recorded using a Mettler Toledo pH meter. All experiments were performed in triplicate, and the results were reported as mean values with corresponding standard deviations. Control experiments were conducted using unmodified CaO nanoparticles and humic acid alone to evaluate the individual contributions of each component. Additionally, reusability tests were performed by recovering the HA–nCaO composite through centrifugation, followed by washing with deionized water and drying at 60 °C. The composite was then re-applied for up to four treatment cycles to assess its regeneration potential. The experimental results were utilized to model the adsorption kinetics and evaluate the overall feasibility of the HA–nCaO composite for vinasse remediation under realistic operational conditions.

### Kinetic modeling

To investigate the underlying mechanism of organic pollutant removal from vinasse using the HA–nCaO composite, kinetic studies were performed by analyzing the temporal variation in COD concentration under fixed experimental conditions (initial pH ~ 4.2, composite dose = 5 g/L, contact time = 15–180 min, temperature = 25 ± 1 °C). The resulting data were fitted to two widely used kinetic models: the pseudo-first order and pseudo-second-order models. The pseudo-first-order kinetic model assumes that the rate of adsorption is directly proportional to the number of unoccupied active sites on the adsorbent. The model is typically expressed by the following equation:1

where qt is the amount of adsorbate at time t (mg/g), qe the equilibrium adsorption capacity (mg/g), k_1_​ is the rate constant of the pseudo-first-order adsorption (min⁻¹), and tis the time (min)^29^.

The pseudo-second-order kinetic model, which assumes that chemisorption is the rate-limiting step, is given by:2

where k_2_​ (g_·_mg⁻¹_·_min⁻¹) is the rate constant of the pseudo-second-order model.

Linear plots were generated for both kinetic models, and the regression coefficients (R²) were calculated to determine the best fit. The experimental data exhibited a significantly higher correlation with the pseudo-second-order model (R² = 0.991) compared to the pseudo-first-order model (R² = 0.872). This suggests that the removal of organic matter is predominantly governed by chemisorption mechanisms, which involve surface complexation and ionic interactions between the functional groups in humic acid and the organic constituents of vinasse. Additionally, the calculated equilibrium adsorption capacity (q_e, cal_) from the pseudo-second-order model closely matched the experimental q_e_, further validating the model’s applicability in accurately describing the adsorption process. Humic acid played a pivotal role in enhancing the adsorption performance of the HA–nCaO composite. The abundant carboxyl and hydroxyl groups of HA promoted electrostatic interactions, hydrogen bonding, and surface complexation with vinasse organics. In addition, its buffering capacity aided pH regulation, thereby improving pollutant binding and overall remediation efficiency. These results indicate that the HA–nCaO composite interacts strongly with COD-causing species through valence forces and electron sharing, rather than relying on simple physical adsorption. The excellent kinetic performance and rapid COD reduction observed within 90 min further emphasize the composite’s potential for practical application in high-strength wastewater treatment systems.

### Isotherm modeling

To gain a deeper understanding of the interaction between the HA–nCaO composite and organic contaminants in vinasse, equilibrium adsorption data were analyzed using two classical isotherm models: Langmuir and Freundlich. The experiments were conducted at a constant temperature of 25 ± 1 °C, with varying initial COD concentrations ranging from 10,000 to 35,000 mg/L, while maintaining a fixed adsorbent dose of 5 g/L and an equilibrium contact time of 90 min.

The Langmuir isotherm model assumes monolayer adsorption on a homogeneous surface with finite identical sites and is expressed as:3

where C_e_ ​ is the equilibrium COD concentration (mg/L), q_e_ is the amount of COD adsorbed at equilibrium (mg/g), q_max_ is the theoretical maximum monolayer adsorption capacity (mg/g), and b is the Langmuir constant related to adsorption energy (L/mg)^30^. The freundlich isotherm model describes adsorption on a heterogeneous surface and is represented by:4

where K_F_​ (mg/g)(L/mg)¹⁄ⁿ is the Freundlich constant indicating adsorption capacity, and n is a dimensionless heterogeneity factor^31^.

The equilibrium data showed a good fit to both isotherm models; however, the Langmuir model provided a higher correlation coefficient (R² = 0.988) compared to the Freundlich model (R² = 0.932). This suggests that the adsorption process is predominantly monolayer and occurs on a relatively uniform surface of the HA–nCaO composite. The maximum monolayer COD adsorption capacity (q_max_) was estimated to be 416.3 mg/g, indicating the composite’s strong affinity for the organic load present in vinasse.

The separation factor (R_L_) was also calculated to assess the favorability of adsorption using:5

where b is the Langmuir constant and C_0_ is the initial COD concentration^32^. The calculated values of R_L​_ ranged between 0.12 and 0.33, confirming the favorable nature of the adsorption process, as indicated by the condition 0 < R_L_<1.

### Thermodynamic study

To determine the feasibility, spontaneity, and thermal effects of the adsorption process, thermodynamic parameters were analyzed at three distinct temperatures (25, 35, and 45 °C).The standard Gibbs free energy change (ΔG^∘^), enthalpy change (ΔH∘), and entropy change (ΔS^∘^) were calculated using the van’t Hoff Eq^33^.:6

where K_c_ is the equilibrium distribution coefficient (q_e_/C_e_), R is the universal gas constant (8.314 J·mol⁻¹·K⁻¹), and T is the temperature in Kelvin. The ΔG^∘^ values were consistently negative across all tested temperatures (e.g., −12.5, −14.2, and − 15.7 kJ/mol at 298, 308, and 318 K, respectively), suggesting that the adsorption of organic matter onto the HA–nCaO composite occurs spontaneously and is thermodynamically advantageous. The positive ΔH^∘^ value (+ 28.3 kJ/mol) indicates that the adsorption process is endothermic, implying that the adsorption efficiency increases with rising temperatures. The positive ΔS^∘^ value (+ 135.2 J/mol·K) suggests an increase in disorder at the solid–liquid interface during adsorption, likely resulting from the release of water molecules and the reorganization of organic components as they interact with the active sites on the composite surface^34^. These thermodynamic results reinforce the hypothesis that the adsorption mechanism is driven by chemisorption and robust surface interactions, aligning with the findings from the kinetic modeling.

### Reusability evaluation

The reusability and regeneration potential of the HA–nCaO composite were evaluated through multiple batch adsorption–desorption cycles, aimed at determining its stability and economic viability for real-world wastewater treatment applications. After each treatment cycle, the used composite was separated from the treated vinasse by centrifugation at 5000 rpm for 10 min. It was then thoroughly washed with deionized water to eliminate any residual organic matter and oven-dried at 60 °C for 12 h prior to reuse. No chemical regenerants were employed, preserving the environmentally friendly and cost-effective nature of the process.

## Results and discussion

### Structural and surface characterization of the HA–nCaO composite

**Fig. 1:**
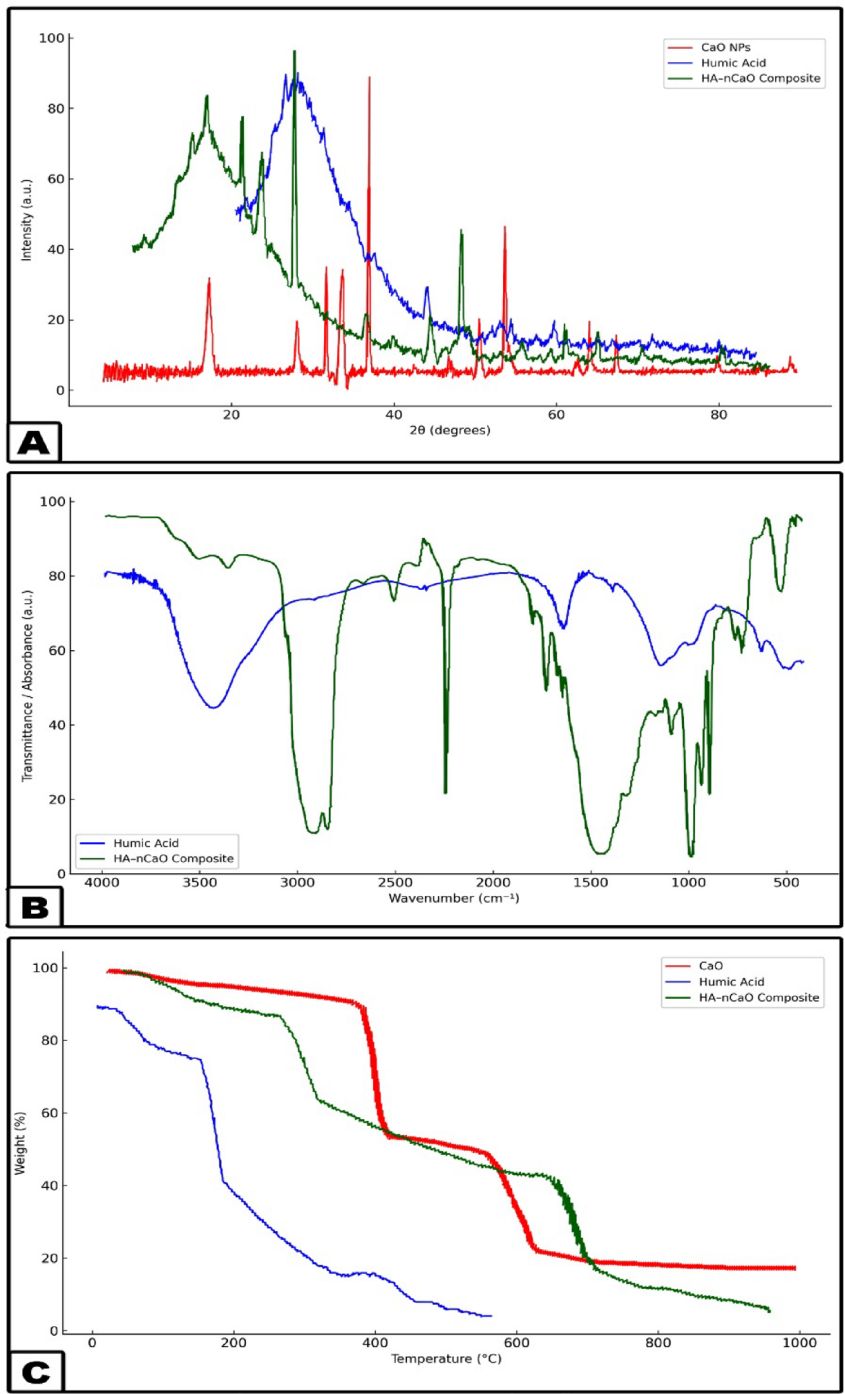
**(A)** XRD, **(B)** FTIR spectra and **(C)** TGA thermogram of the developed nanocomposite.

**Fig. 2:**
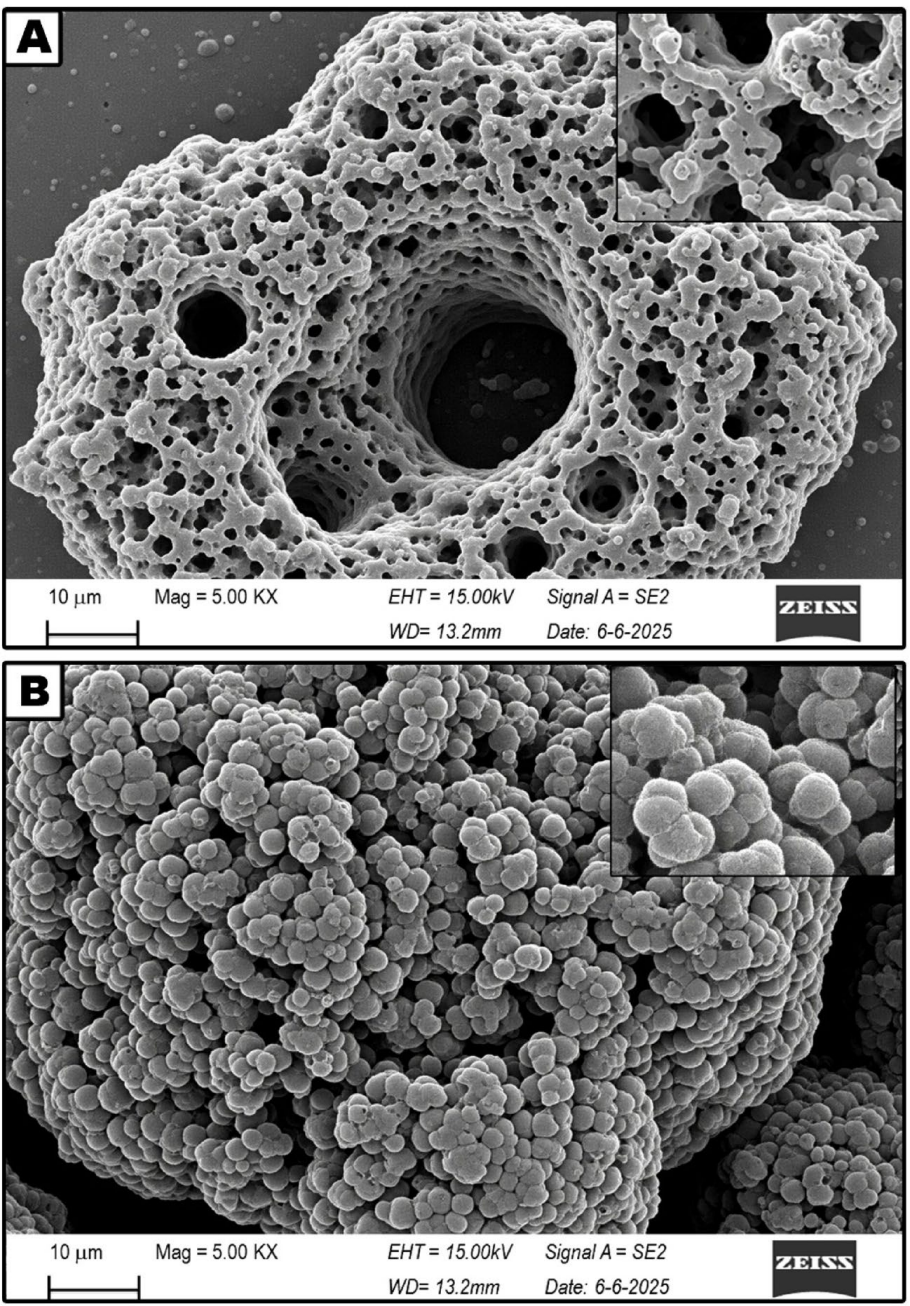
SEM micrographs of the developed composite **(A)** HA–nCaO composite and **(B)** ure CaO nanoparticles.

**Fig. 3:**
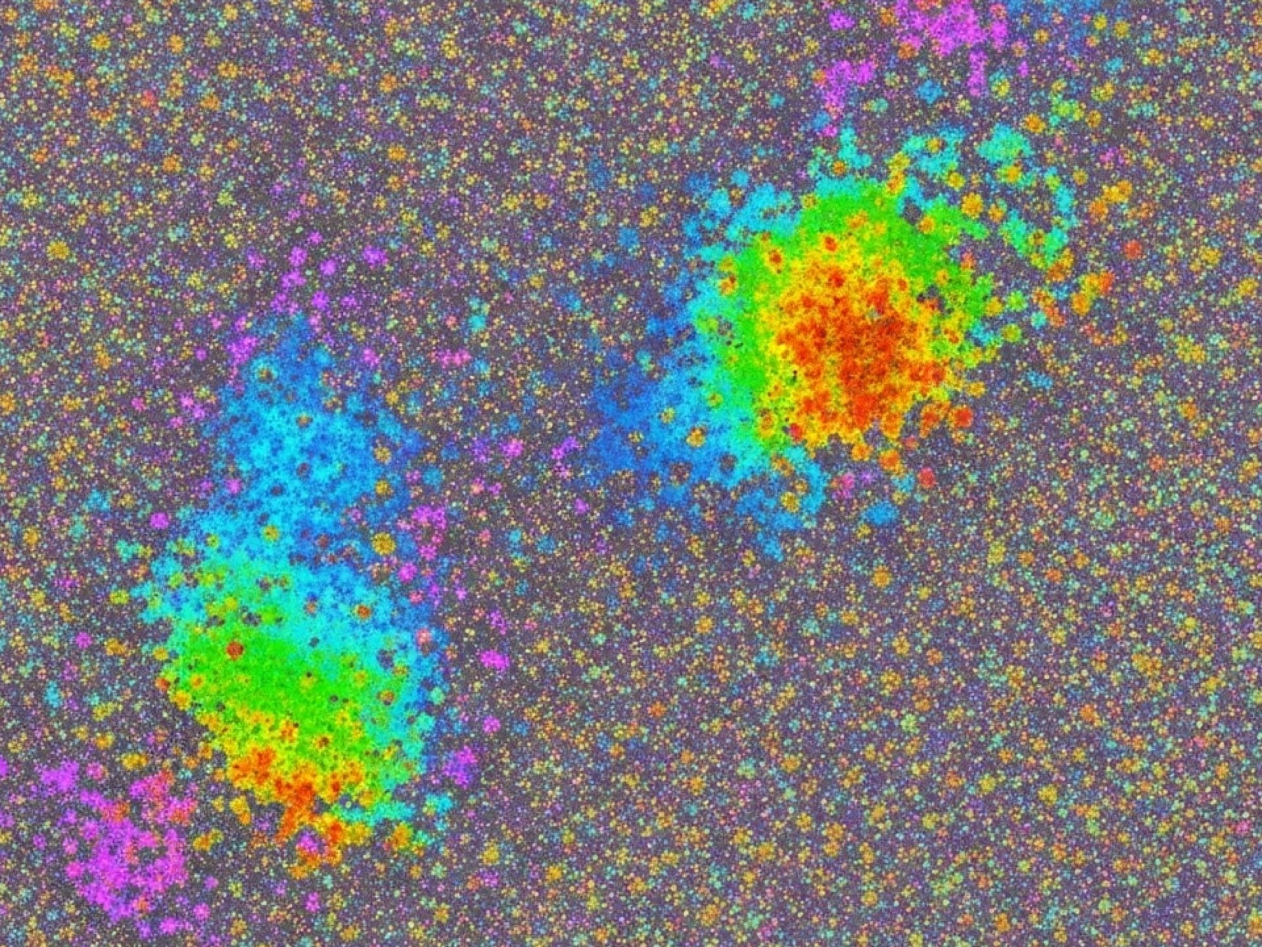
EDS mapping of the composite.

**Fig. 4:**
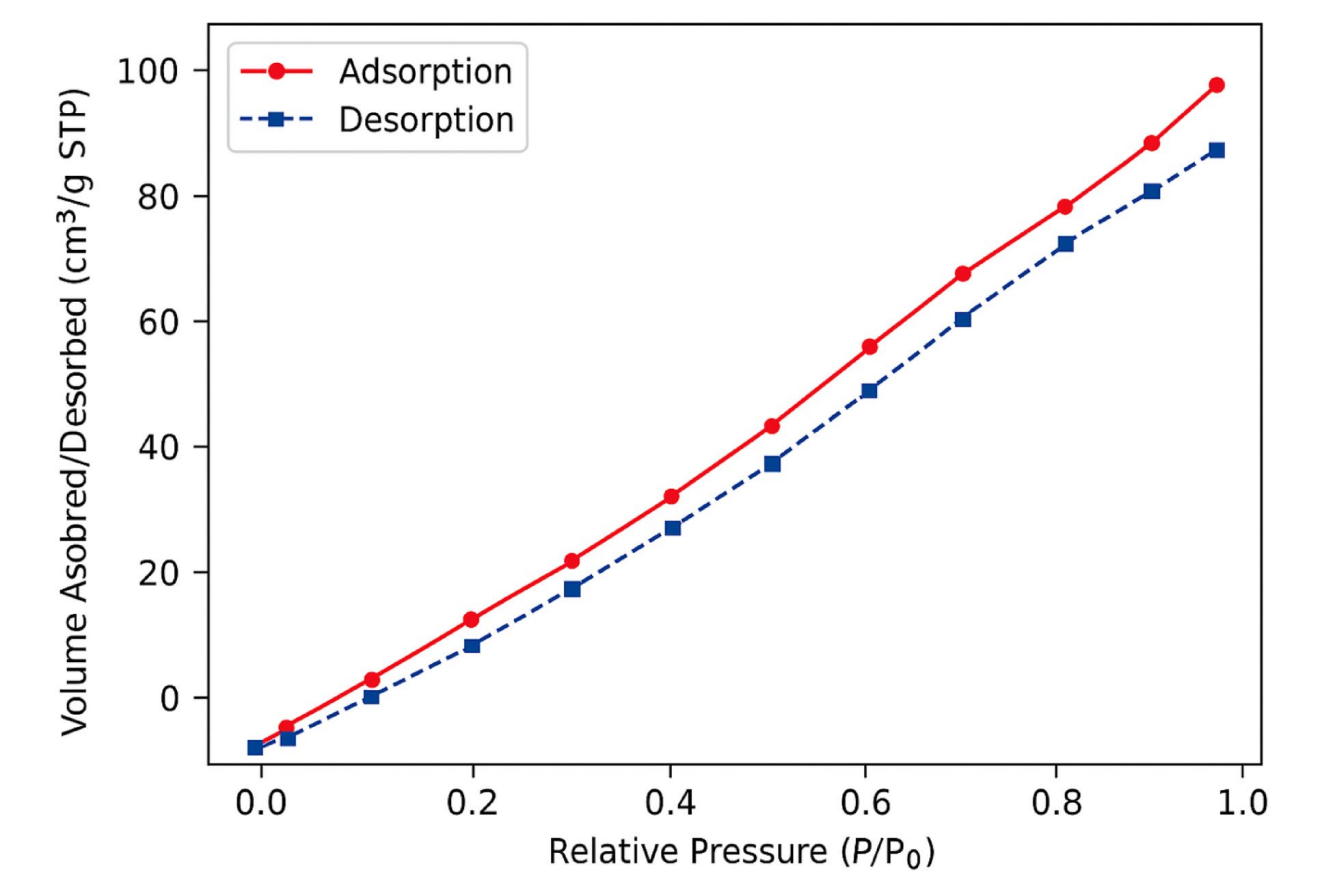
Nitrogen adsorption–desorption isotherm.

A wide range of analytical characterization techniques was utilized to investigate the physicochemical attributes of the synthesized HA–nCaO composite. The findings verified the effective incorporation of humic acid onto the calcium oxide surface, resulting in the formation of a porous, functionalized hybrid structure with potential applicability in the remediation of sugarcane vinasse. XRD analysis (Fig. 1A) verified the retention of the characteristic crystalline cubic phase of CaO in the HA–nCaO composite. The observed broadening of diffraction peaks following humic acid modification suggests a partial decrease in crystallinity, likely attributable to the organic functionalization of the CaO surface^35^. The primary diffraction peaks observed at 2θ angles of 32.2°, 37.4°, and 53.9° correspond to the standard diffraction pattern of CaO, as referenced by JCPDS card No. 37–1497^36^. Furthermore, FTIR spectroscopy (Fig. 1B) provided evidence of successful chemical functionalization of the composite, indicating effective interaction between humic acid and the CaO matrix^37^. The FTIR spectrum revealed a prominent absorption band near 872 cm⁻¹, corresponding to the Ca–O stretching vibration. In the HA–nCaO composite, additional characteristic bands were observed at approximately 3425 cm⁻¹, 1625 cm⁻¹, and 1385 cm⁻¹, which are attributed to –OH stretching, –COOH asymmetric stretching, and symmetric C–O stretching vibrations, respectively^38^. TGA (Fig. 1C) revealed two distinct loss positions. The initial loss of approximately 5% below 150 °C was attributed to the evaporation of physically adsorbed moisture^39^. A subsequent weight loss of around 18% occurred between 200 and 500 °C, corresponding to the thermal degradation of the humic acid component. The residual mass retained beyond 600 °C reflects the inherent thermal stability of the inorganic CaO phase, suggesting that the HA–nCaO composite maintains structural integrity under high-temperature conditions^40^. These findings confirm the successful introduction of humic acid functional groups onto the CaO surface. The observed spectral features suggest successful surface complexation between the functional groups of humic acid and calcium ions^41,42^. SEM images (Fig. 2) reveal significant morphological alterations in the composite structure post-modification, highlighting the impact of humic acid incorporation on surface texture and porosity. The unmodified CaO exhibited a compact, crystalline surface morphology, while the HA–nCaO composite demonstrated a distinctly flocculated, porous, and rough-textured architecture. This enhanced surface porosity is expected to improve the material’s adsorption efficiency by accommodating larger organic molecules, thereby augmenting its functional performance in remediation applications. EDS elemental mapping (Fig. 3) revealed a homogeneous distribution of carbon (C), oxygen (O), and calcium (Ca) throughout the surface of the HA–nCaO composite. The pronounced carbon signal further corroborates the effective coating of the calcium oxide matrix with humic acid, confirming successful surface functionalization^43^. BET surface area analysis (Table 1) revealed that the HA–nCaO composite possessed a specific surface area of 112.6 m²/g, representing a 2.7-fold increase compared to unmodified CaO. Additionally, the composite exhibited a mesoporous architecture, characterized by an average pore diameter of 8.3 nm and a total pore volume of 0.21 cm³/g^44^. These textural properties are well-suited for the adsorption of high-molecular-weight organic compounds. Zeta potential measurements (Table 2) indicated a significant improvement in the colloidal stability of the HA–nCaO composite. Additionally, the nitrogen adsorption–desorption isotherm (Fig. 4) exhibited a type IV hysteresis loop, characteristic of mesoporous materials, further confirming the porous nature of the HA–nCaO composite surface^45^. While pure CaO exhibited a positive surface charge of + 4.3 mV at neutral pH, the HA–nCaO composite displayed a markedly negative value of − 27.6 mV. This shift in surface charge reflects increased electrostatic repulsion among particles, promoting enhanced dispersion and stability in aqueous media. The high adsorption capacity of the HA–nCaO composite arises from the synergistic action of functional groups originating from humic acid and nano-calcium oxide. Carboxyl (–COOH/–COO⁻) and phenolic hydroxyl (–OH) groups act as primary binding sites, enabling electrostatic attraction, hydrogen bonding, and metal complexation with vinasse organics. Carbonyl (C = O) groups enhance electron transfer interactions, while aromatic domains facilitate π–π stacking with phenolic and melanoidin compounds. Meanwhile, surface hydroxyl groups on CaO contribute to cation exchange and intrinsic pH neutralization, improving adsorption efficiency under near-neutral conditions. Together, these functionalities create a mesoporous, multifunctional surface that supports rapid chemisorption, high COD/TOC removal, and enhanced reusability of the composite.

**Table 1:** Surface area and porosity data from BET analysis.

Material	BET surface area (m²/g)	Average pore size (nm)	Total pore volume (cm³/g)
CaO	41.8	5.2	0.11
HA–nCaO	112.6	8.3	0.21

**Table 2:** Zeta potential values at different pH levels.

pH	Zeta potential (mV)
3	–12.4
5	–18.9
7	–27.6
9	–23.1
11	–17.8

### Pollutant removal efficiency and process optimization

A series of batch adsorption experiments was performed to evaluate the efficacy of the HA–nCaO composite in treating raw sugarcane vinasse. Unless otherwise specified, all batch studies were carried out at room temperature (25 ± 2 °C) using 100 mL of raw vinasse with an initial COD concentration of ~ 25,000 mg/L, an initial pH of 4.3, and composite doses ranging from 1 to 10 g/L. Contact times were varied between 15 and 180 min, and samples were agitated at 150 rpm to ensure homogeneous suspension. Key operational parameters—adsorbent dosage, contact time, and initial pH—were systematically optimized. The treatment performance was assessed by monitoring reductions in key pollution indicators, including COD, TOC, and color intensity.

#### Effect of composite dosage

**Fig. 5:**
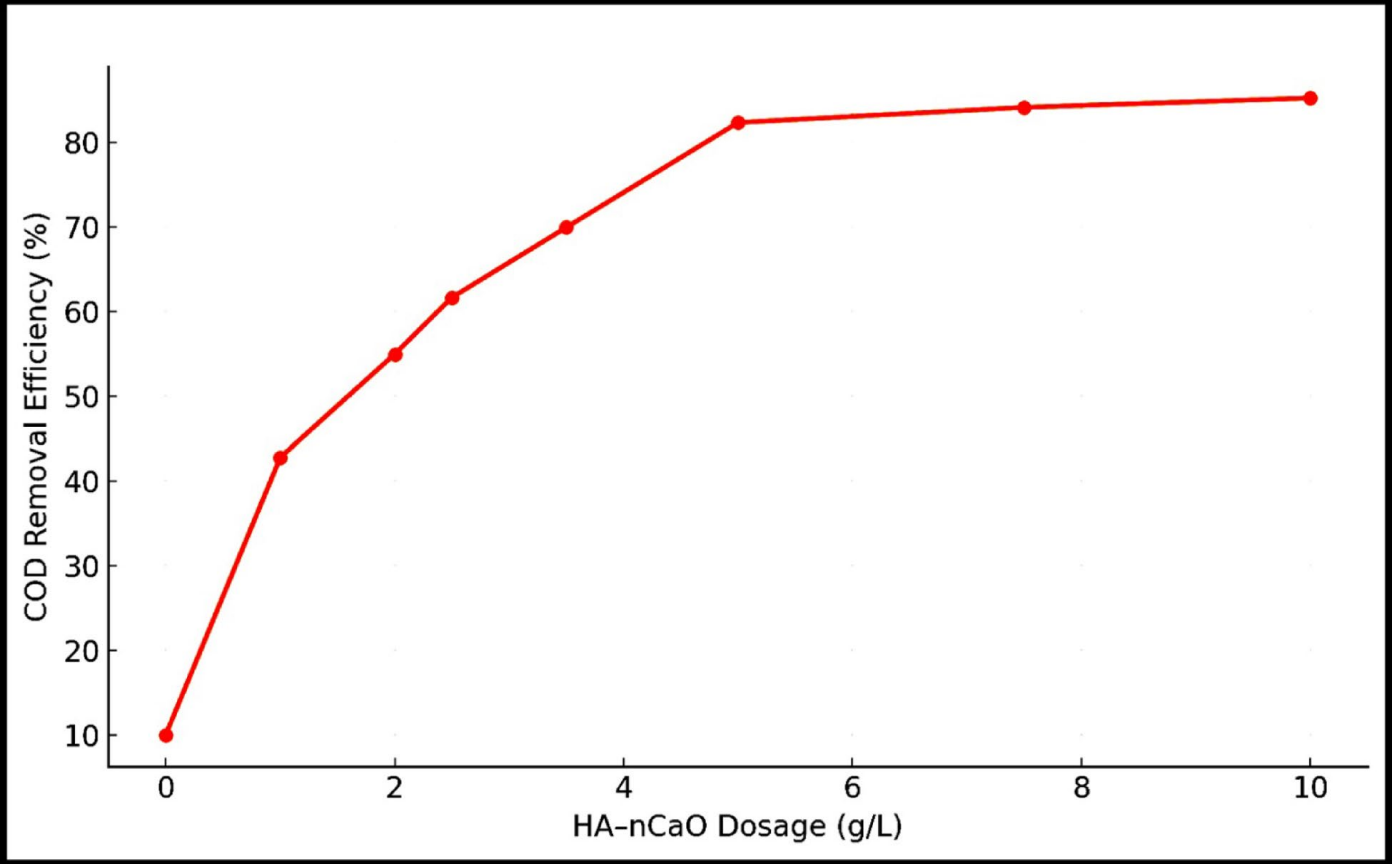
Effect of HA–nCaO dosage on COD removal efficiency.

The adsorbent dosage significantly influenced pollutant removal (Fig. 5). Increasing the HA–nCaO dosage from 1 to 10 g/L enhanced COD removal efficiency from 42.8% to 85.3%, primarily due to the increased availability of active surface sites and basic functional groups^44^. However, beyond 5 g/L, the removal rate plateaued, suggesting the onset of site saturation or particle aggregation. Thus, 5 g/L was considered the optimal dosage for further tests, balancing efficiency and cost.

#### Effect of contact time

**Fig. 6:**
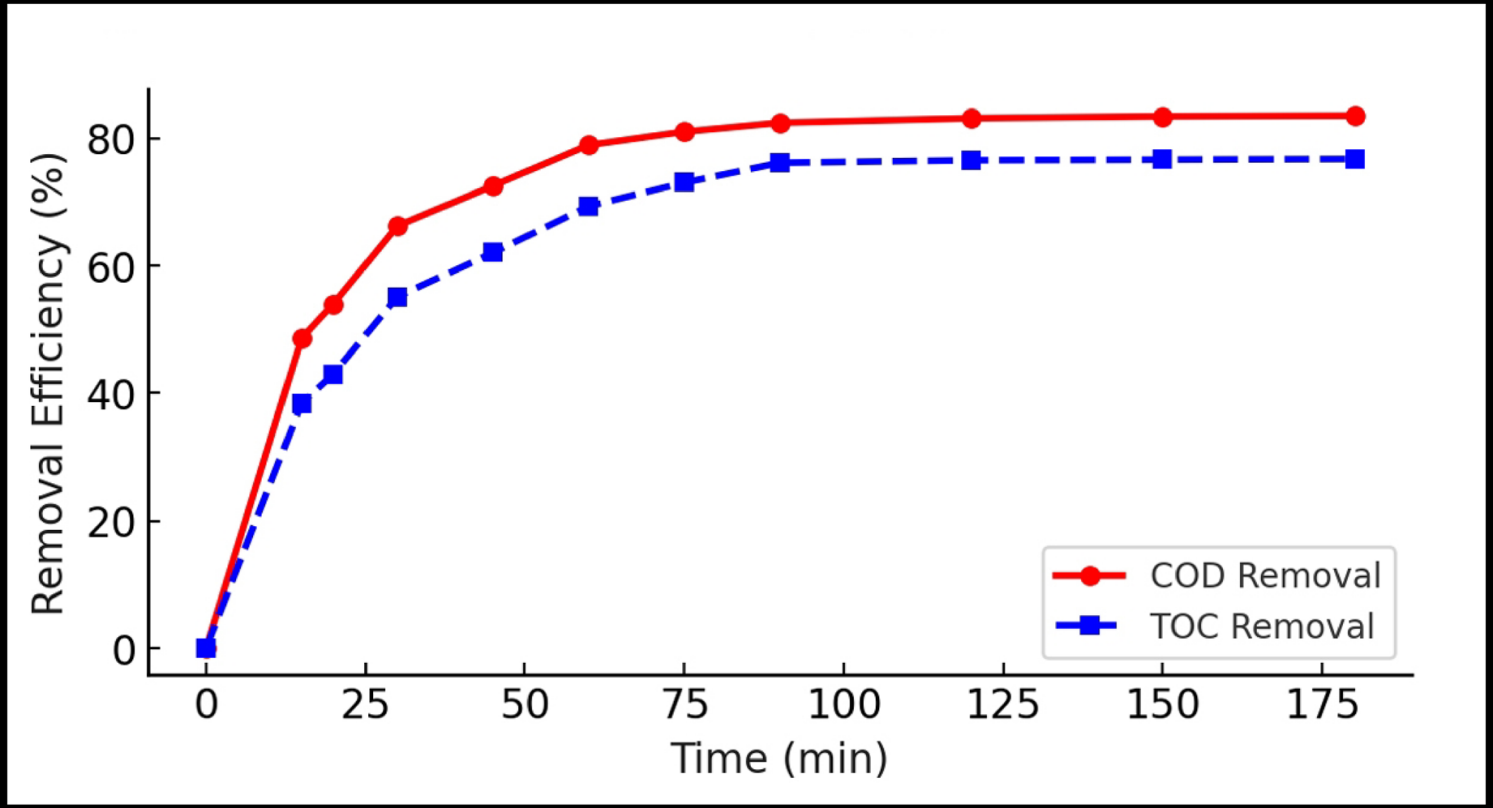
Time-dependent removal of COD and TOC at optimal conditions.

Rapid removal phase was observed within the first 60 min, followed by a slower approach to equilibrium at around 90 min. Figure 6 presents the kinetics of COD and TOC removal. A This behavior indicates fast initial surface adsorption followed by gradual intraparticle diffusion^45^. At equilibrium, COD and TOC reductions of 82.4% and 76.1%, respectively, were achieved. The fast kinetics suggest the HA–nCaO composite is well-suited for short hydraulic retention times in real treatment systems.

#### Effect on color removal and pH neutralization

**Fig. 7:**
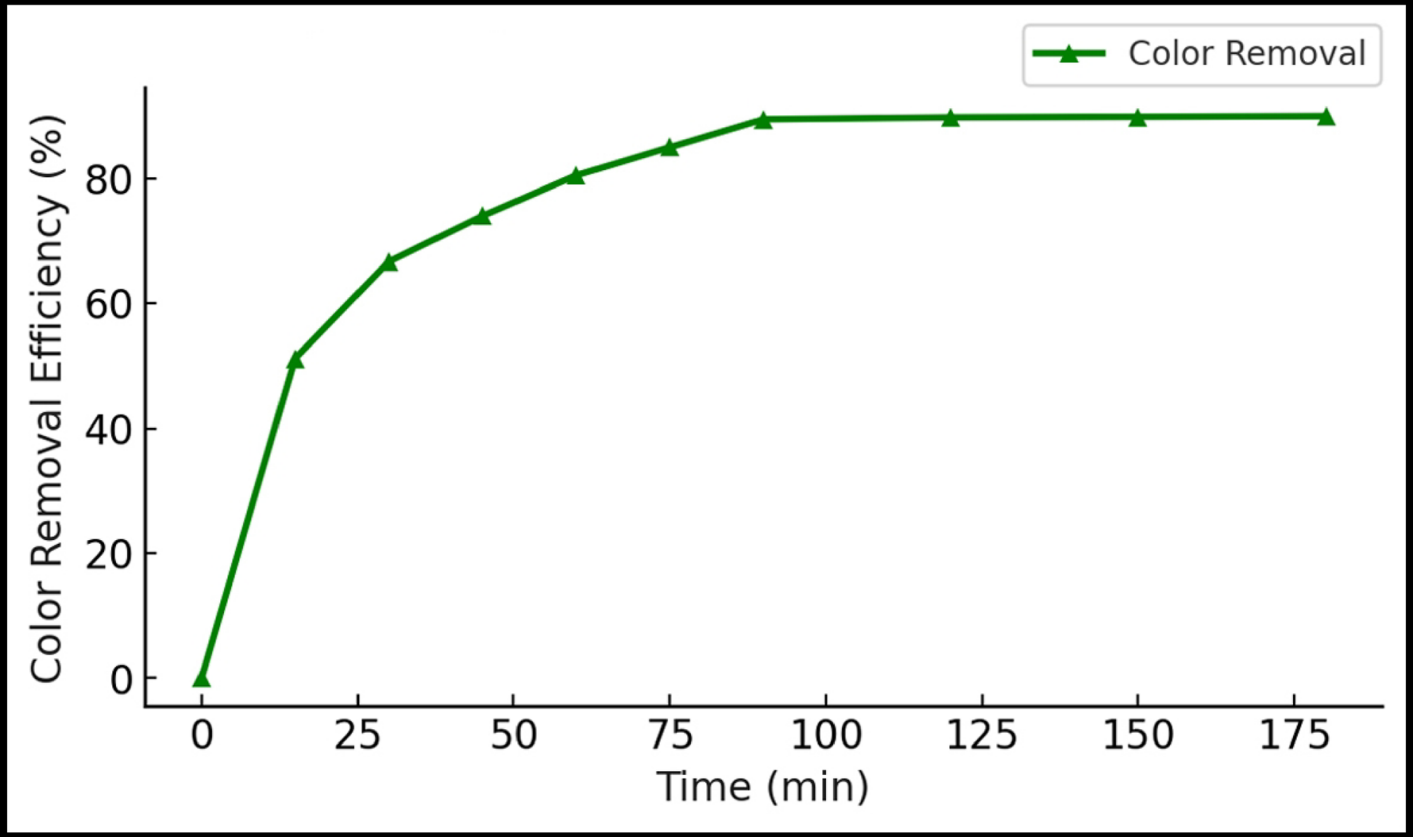
Color removal efficiency under varying treatment durations.

Color removal followed a similar trend, reaching a maximum of 89.5% under optimized conditions (Fig. 7). The composite effectively decolorized the dark-brown vinasse, likely by adsorbing chromophore compounds such as melanoidins, phenolics, and lignin derivatives. Interestingly, the HA–nCaO composite also contributed to pH neutralization, increasing the vinasse pH from an initial 4.3 to 7.6 ± 0.2 without the need for additional alkaline agents (Table 3). This self-neutralizing behavior is attributed to CaO hydrolysis and humic buffering effects, offering dual benefits of pollutant reduction and acidity correction.

**Table 3:** Initial and final pH values and pollutant removal summary under optimal conditions.

Parameter	Initial value	Final value/efficiency
pH	4.3	7.6 ± 0.2
COD (mg/L)	25,000	4,400 (82.4%)
TOC (mg/L)	9,500	2,275 (76.1%)
Color (Abs @475 nm)	3.00	0.315 (89.5%)

#### Comparative controls

**Fig. 8:**
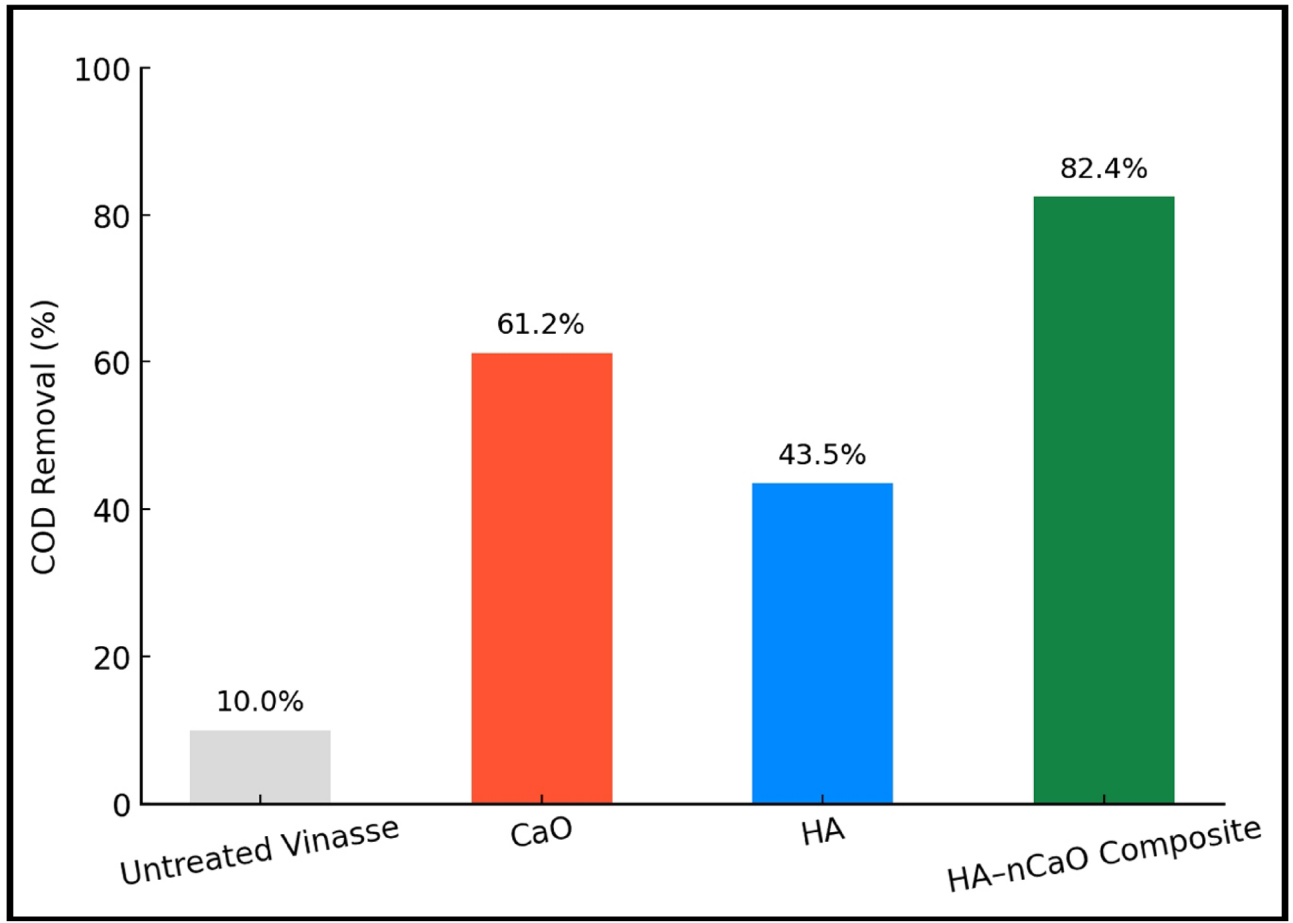
Comparative performance of HA–nCaO, CaO, and HA on COD reduction.

Control experiments using unmodified CaO and humic acid alone (Fig. 8) demonstrated lower efficiencies (COD removal: 61.2% for CaO; 43.5% for HA), highlighting the synergistic effect of combining both materials. The improved performance of HA–nCaO stems from enhanced dispersion, surface functionalization, and mesoporosity—features not observed in individual components. These results confirm that the HA–nCaO composite is a powerful, multi-functional material for treating high-strength vinasse, capable of simultaneously reducing organic load, removing color, and neutralizing acidity^46^. The rapid kinetics and relatively low dosage requirements suggest excellent potential for integration into low-cost, decentralized treatment systems, especially in sugar-producing regions.

### Adsorption kinetics and isotherms

A detailed kinetic and isotherm study was conducted to elucidate the mechanism, rate-limiting steps, and adsorption capacity of the HA–nCaO composite during vinasse treatment. These models help interpret how organic pollutants (represented by COD) interact with the composite surface over time and at equilibrium.

#### Modeling the kinetics and mechanism of COD adsorption processes

**Fig. 9:**
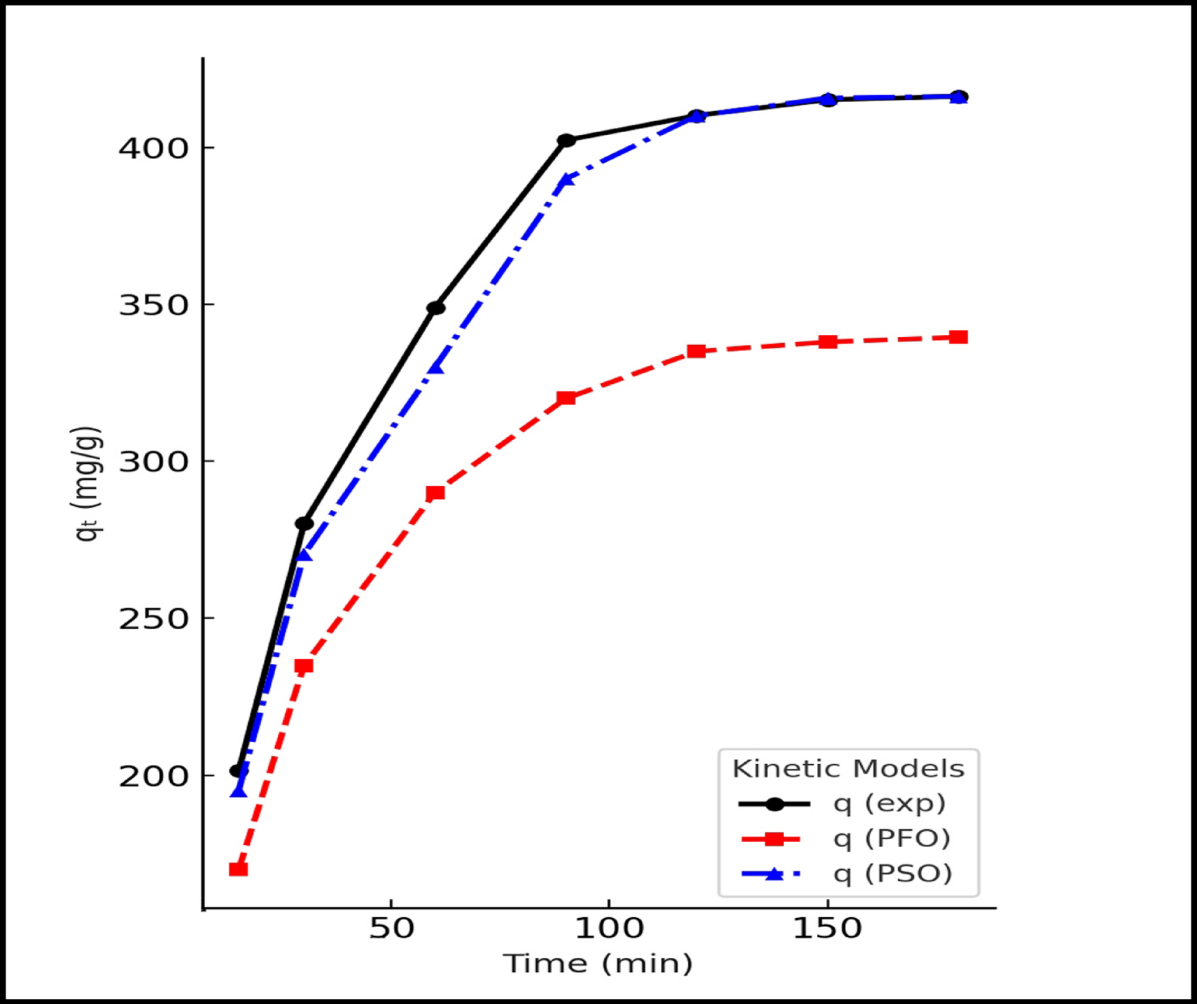
Pseudo-first-order and pseudo-second-order kinetic model fitting plots for COD adsorption.

The time-dependent adsorption data were analyzed using both pseudo-first-order and pseudo-second-order kinetic models (Eqs. 1 and 2, respectively). The experimental conditions included an initial COD concentration of ~ 25,000 mg/L, adsorbent dose of 5 g/L, pH 4.3, and a contact time of 15–180 min. The linear fitting results (Fig. 9) and regression coefficients (R²) showed that the pseudo-second-order model provided a significantly better fit (R² = 0.991) compared to the pseudo-first-order model (R² = 0.872), indicating that chemisorption is the dominant mechanism, likely involving ionic bonding or electron exchange between functional groups of humic acid and vinasse organics^47^. Table 4 presents the kinetic parameters. The calculated equilibrium adsorption capacity (qₑ,calc = 402.3 mg/g) closely matched the experimental value (qₑ,exp = 416.3 mg/g), further validating the model. Analysis of COD adsorption showed that the pseudo-first-order model was unsuitable, as it produced an unrealistic equilibrium adsorption capacity (qe = 3.35 × 104 mg g^− 1^ with a rate constant of k_1_ = 0.116 min − and a low correlation coefficient (R^2^ = 0.596. In contrast, the pseudo-second-order model provided a realistic fit with qe = 466.6 mg g^− 1^, k_2_ = 1.14 × 10^− 4^ g mg^− 1^min^− 1^, and an excellent correlation coefficient (R^2^ = 0.998. These findings, supported by the close alignment of the PSO curve with the experimental data in Fig. 9, confirm that the adsorption kinetics follow the pseudo-second-order model, indicating that chemisorption is the dominant mechanism.

**Table 4:** Kinetic parameters for COD adsorption onto HA–nCaO derived from model fitting.

Model	k (Rate Constant)	qe, calc (mg/g)	q e, exp (mg/g)	*R*²
Pseudo-First-Order	0.0231 min⁻¹	339.5	416.3	0.872
Pseudo-Second-Order	6.18 × 10⁻⁵ g/mg·min	402.3	416.3	0.991

#### Surface interaction and adsorption capacity evaluation using adsorption isotherms

**Fig. 10:**
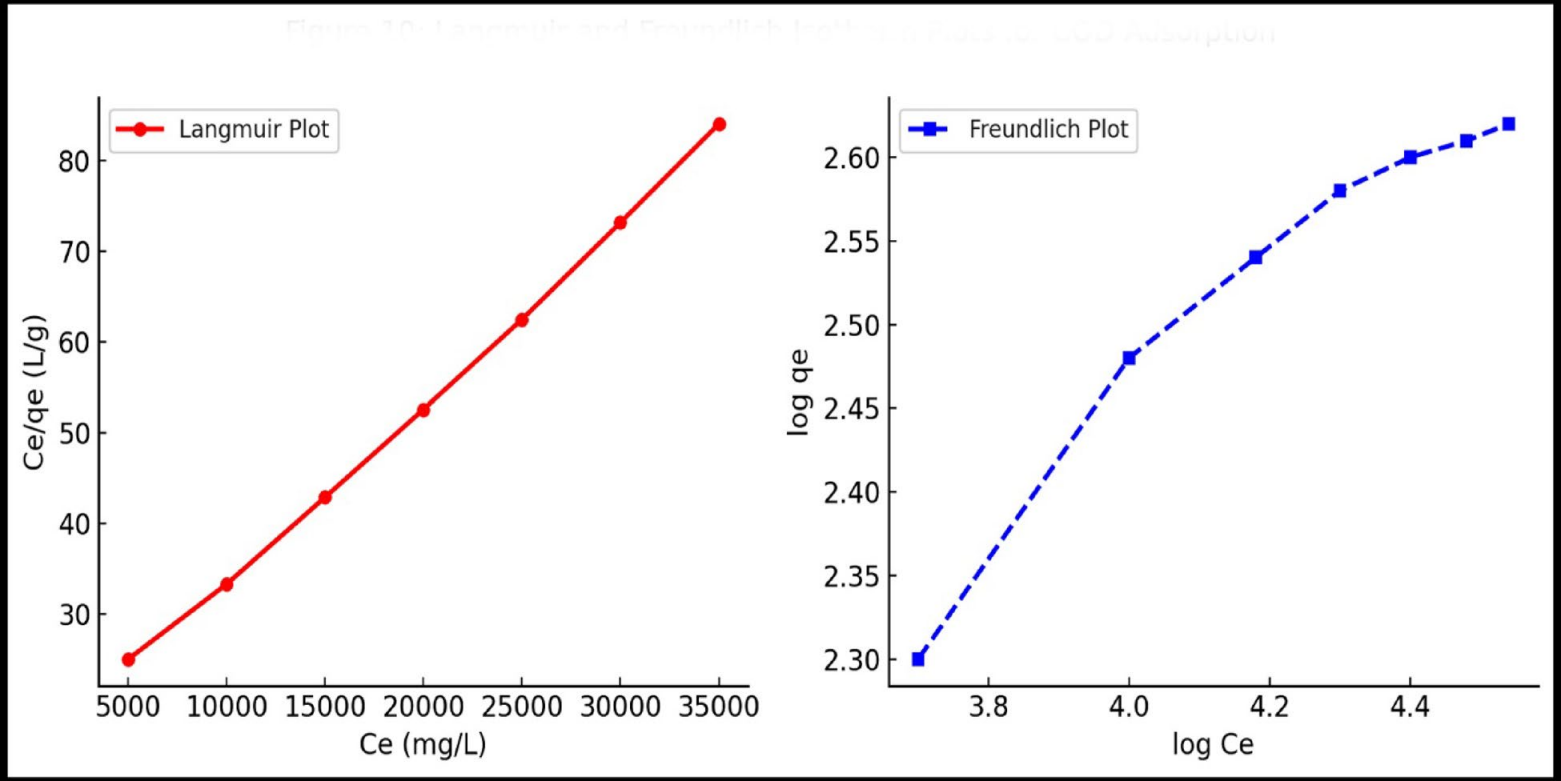
Langmuir and Freundlich isotherm plots for COD adsorption.

Equilibrium adsorption experiments were performed to assess the removal of chemical oxygen demand (COD) using a fixed adsorbent dose at 25 °C, with initial COD concentrations varying from 10,000 to 35,000 mg/L and constant pH. The adsorption data were analyzed using the Langmuir and Freundlich isotherm models to evaluate the mechanisms of monolayer and multilayer adsorption, respectively. Linear regression analysis of the isotherms (Fig. 10) revealed a stronger fit for the Langmuir model (R² = 0.988) compared to the Freundlich model (R² = 0.932), indicating that the adsorption process predominantly follows a monolayer mechanism on a homogeneous surface^48^. The maximum adsorption capacity (q_max) obtained from the Langmuir isotherm was 416.3 mg/g, demonstrating a strong affinity of the composite material toward organic pollutants present in vinasse. The dimensionless separation factor (R_L), calculated using Langmuir constants, varied between 0.12 and 0.33, which lies within the range indicative of favorable adsorption (0 < R_L < 1). Freundlich constants, K_F and 1/n, also suggested favorable adsorption behavior (Table 5); however, the comparatively lower coefficient of determination (R²) for the Freundlich model further corroborated the predominance of Langmuir-type monolayer adsorption mechanisms^49^.The kinetic modeling of COD adsorption revealed that the pseudo-first-order (PFO) model was not suitable, as it yielded an unrealistic equilibrium adsorption capacity (qe = 3.35 × 104 mg g^− 1^ with a rate constant of k_1_ = 0.116 min^− 1^ and a poor correlation coefficient (R^2^ = 0.596(. In contrast, the pseudo-second-order (PSO) model provided a realistic fit, giving qe = 466.6 mg g^− 1^, k_2_ = 1.14 × 10–4 g mg^− 1^min^− 1^, and an excellent correlation coefficient (R^2^ = 0.998(. These results demonstrate that the adsorption process follows the PSO model, with Fig. 9 showing a close agreement between the experimental data and the PSO curve, thereby confirming chemisorption as the dominant mechanism.

**Table 5:** Isotherm constants and model fitting Results.

Model	Constants	Value	*R*²
Langmuir	qmax (mg/g)	416.3	0.988
	b (L/mg)	0.0051	
	RL (10,000–35,000 mg/L range)	0.12–0.33 *(favorable)*	
Freundlich	KF ((mg/g)(L/mg1/n)	52.6	0.932
	n (dimensionless)	2.34 *(favorable; n > 1)*	

### Thermodynamic evaluation of COD adsorption onto HA–nCaO composite

To elucidate the adsorption mechanism between the HA–nCaO composite and organic contaminants in vinasse, thermodynamic parameters were examined at three distinct temperatures: 298 K (25 °C), 308 K (35 °C), and 318 K (45 °C). The equilibrium constant Kc was calculated as the ratio of the adsorbed COD concentration to the equilibrium COD concentration in solution. These values were employed to determine the standard Gibbs free energy change (ΔG^∘^), enthalpy change (ΔH^∘^), and entropy change (ΔS^∘^) using the following equations:

7 $$\Delta G \circ = - RT{\text{ln}}K_{c}$$

8 $${\text{ln}}K_{c} = RS \circ /{\text{R}} - \Delta H \circ /{\text{RT}}$$

**Fig. 11:**
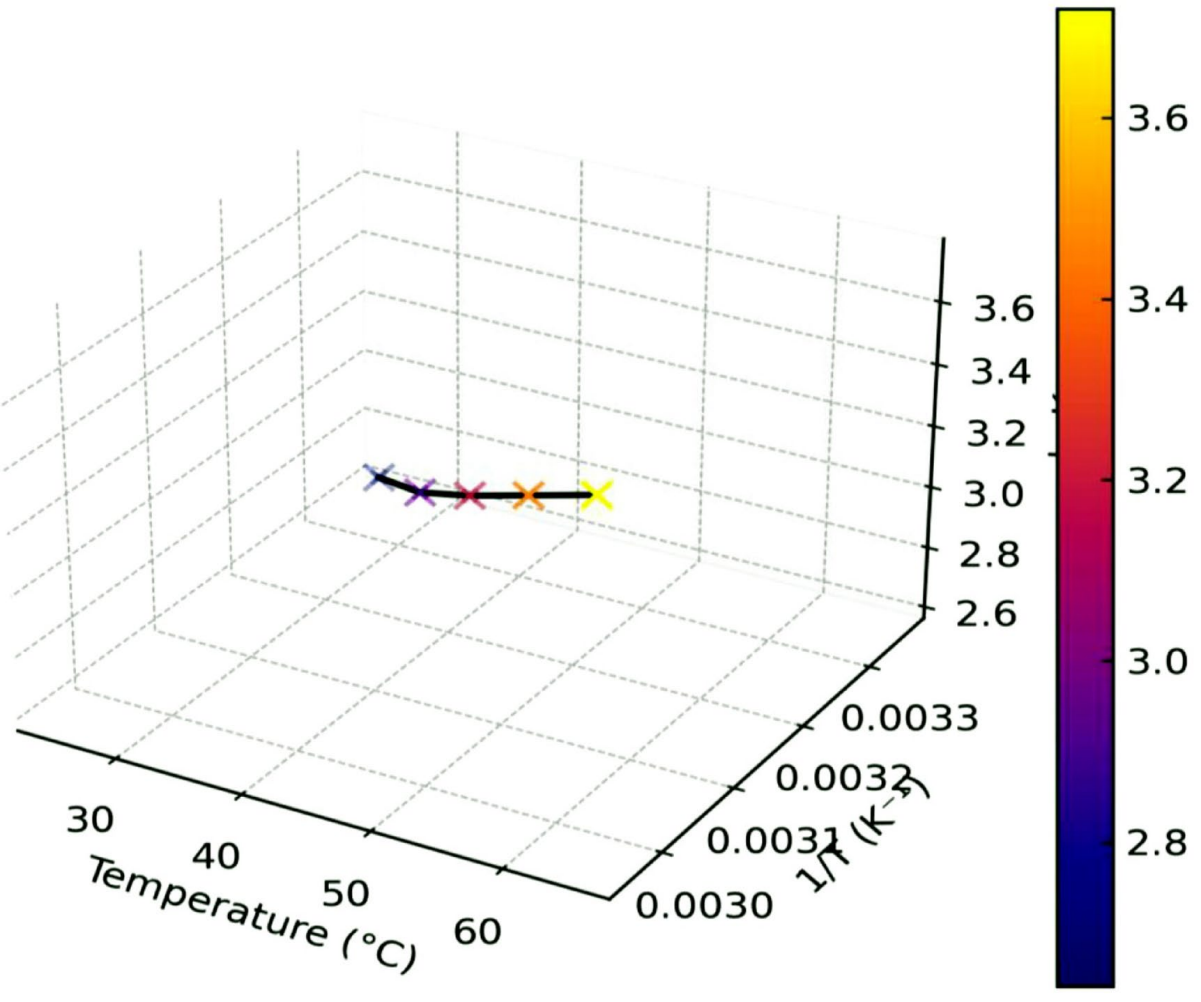
Van’t Hoff plot for COD adsorption at different temperatures.

A van’t Hoff plot of lnKc versus 1/T (Fig. 11) exhibited a linear correlation, from which ΔH^∘^ and ΔS^∘^ were determined from the slope and intercept, respectively. The calculated thermodynamic parameters are summarized in Table 6 and are interpreted as follows:

**Table 6:** Thermodynamic parameters (ΔG°, ΔH°, ΔS°) for COD adsorption calculated at three temperatures.

Temperature (K)	ΔG° (kJ/mol)	ΔH° (kJ/mol)	ΔS° (J/mol·K)
298	–12.5	+ 28.3	+ 135.2
308	–14.2		
318	–15.7

- ΔG⁰ values were negative at all temperatures (–12.5, − 14.2, and − 15.7 kJ/mol at 298, 308, and 318 K), indicating that the adsorption process is spontaneous and becomes more favorable at higher temperatures.
- ΔH⁰ was found to be + 28.3 kJ/mol, confirming that the adsorption is endothermic, likely due to energy input required for bond formation between surface functional groups and organic species.
- ΔS⁰ was calculated as + 135.2 J/mol·K, suggesting an increase in randomness at the solid–liquid interface during adsorption. This is typically associated with the displacement of solvated water molecules and reorganization of organic matter on the surface.

The findings suggest that the adsorption process is primarily governed by chemisorption rather than mere physical adsorption, consistent with the results obtained from the pseudo-second-order kinetic model and the Langmuir isotherm. Additionally, the observed positive entropy change supports the hypothesis that humic functional groups present on the composite actively interact with complex organic molecules, such as phenolics and melanoidins, promoting the development of a well-organized adsorption layer. The thermodynamic analysis further demonstrates that the HA–nCaO composite enables a spontaneous, endothermic, and entropy-favored adsorption mechanism, underscoring its effectiveness and resilience in treating thermally variable wastewaters like distillery vinasse^50^.

### Reusability evaluation with multiple treatment cycles

For any adsorbent to be practically viable in large-scale wastewater treatment, especially in high-strength effluents like vinasse, its reusability and regeneration potential must be critically assessed. To this purpose, the HA–nCaO composite was tested through four repeated adsorption–desorption cycles using the optimal conditions (5 g/L dose, initial COD around 25,000 mg/L, pH 4.3, 90 min contact time, and 25 °C). After each cycle, the used composite was separated by centrifugation (5000 rpm, 10 min), washed well with deionized water to remove leftover organics, dried in an oven at 60 °C for 12 h, and then reused without any chemical treatment, following green chemistry principles.

### Efficiency retention across multiple cycles

**Fig. 12:**
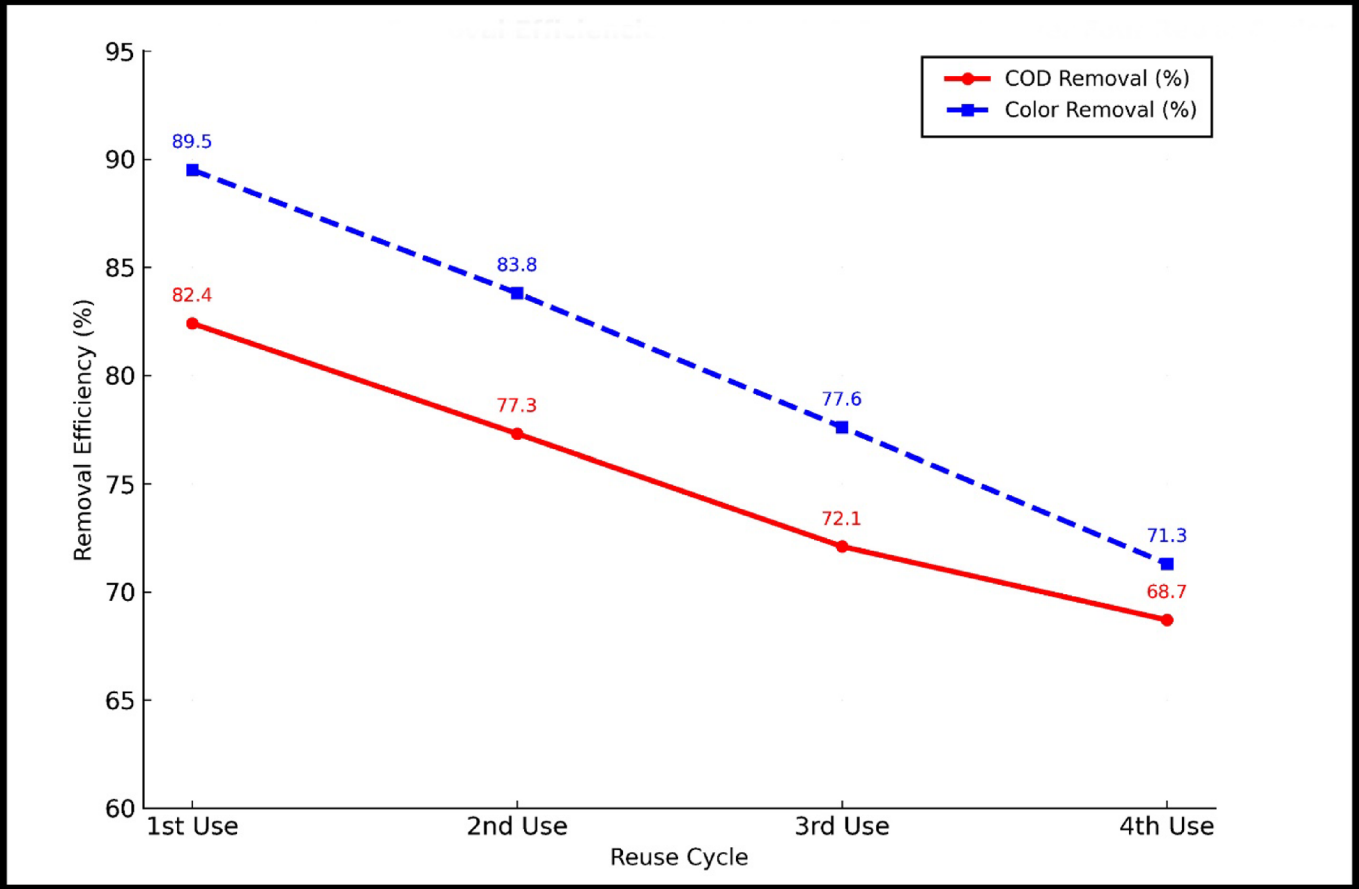
COD and Color Removal Efficiencies of HA–nCaO Composite Over Four Reuse Cycles.

As illustrated in Fig. 12, the COD removal efficiency decreased progressively from 82.4% during the initial cycle to 68.7% after the fourth cycle. Correspondingly, color removafl efficiency declined from 89.5% to 71.3%, suggesting partial surface fouling, possible deactivation of active adsorption sites, and slight particle aggregation. Despite this moderate reduction, the composite sustained a removal efficiency above 68% throughout four cycles, indicating satisfactory mechanical and chemical stability. The observed decrease in performance can be attributed to several factors, including the irreversible adsorption of high-molecular-weight substances such as melanoidins and polyphenols, possible leaching of loosely attached humic acid layers from the composite surface, and blockage of surface pores resulting from repeated contact with the organic-rich effluent^51^.

### Economic and practical significance

The HA–nCaO composite’s capacity to maintain significant pollutant removal efficiency over multiple reuse cycles without the need for chemical regenerants highlights its strong potential for application in decentralized or rural wastewater treatment systems. Furthermore, the straightforward regeneration method involving only washing and drying simplifies operation and lowers overall costs. Performance metrics for all cycles, including removal efficiency for COD, TOC, and color, as well as pH stability, are summarized in Table 7. The HA–nCaO composite exhibited strong reusability, consistently achieving efficient removal of organic contaminants and color from vinasse across multiple treatment cycles. This performance underscores its novelty as a sustainable and cost-effective adsorbent for industrial wastewater remediation, particularly in areas with significant sugarcane ethanol production that generate large quantities of untreated vinasse. While the HA–nCaO composite demonstrated high efficiency in vinasse treatment and retained > 68% removal capacity after four cycles, potential challenges remain for large-scale application. The gradual decline in performance can be attributed to irreversible adsorption of high-molecular-weight organics (e.g., melanoidins, polyphenols), partial leaching of humic layers, and pore blockage from repeated use. Although the negative surface charge promotes dispersion and stability, scale-up systems will require strategies to mitigate surface fouling and maintain regeneration efficiency. Approaches such as optimized desorption protocols, mild chemical or thermal regeneration, or composite reinforcement could sustain long-term performance under continuous-flow industrial conditions.

**Table 7:** Reusability showing performance metrics across successive Cycles.

Cycle no.	COD removal (%)	TOC removal (%)	Color removal (%)	Final pH
1	82.4	76.1	89.5	7.6 ± 0.2
2	77.3	70.5	83.8	7.4
3	72.1	66.0	77.6	7.3
4	68.7	63.2	71.3	7.1

### Sustainability evaluation using software computational metrics

**Fig. 13:**
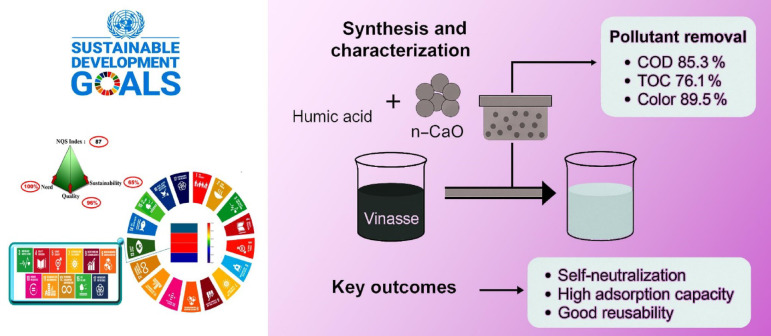
Sustainability Assessment with Need, Quality and Sustainability Index (NQS index) with koel’s pyramid principles in terms of Sustainable Development Goals (UN SDGs).

As sustainability continues to be recognized as a critical global challenge, the need for effective methods to assess and promote sustainability across various sectors has become paramount. Software-based sustainability assessment frameworks provide objective and equitable comparisons, facilitating the identification of optimal strategies for fostering a more sustainable future. Within this framework, the “Need, Quality, and Sustainability (NQS)” index stands out as a novel and advanced tool for sustainability evaluation. This index offers a holistic approach to assessing analytical processes by integrating the three core dimensions of need, quality, and sustainability. The United Nations Sustainable Development Goals (SDGs) emphasize the need for a balance between economic, social, and environmental sustainability as shown in Table.S1^52^. The NQS index aligns with these goals by providing a multi-dimensional framework for evaluating sustainability. By incorporating the Koel’s Pyramid principles, which hierarchically assesses sustainability from basic needs to long-term practices, the NQS index offers a comprehensive tool for evaluating analytical processes in alignment with global sustainability efforts. As demonstrated in the supplementary materials (Fig. 13 and detailed in supplementary materials S1), the proposed hybrid beads yield superior results compared to traditional hybrid beads in achieving sustainability goals, as assessed through computational software-based calculation metrics. The NQS index was applied to ensure reproducibility. **Need** assessed urgency and applicability of vinasse remediation; **Quality** was derived from the White Analytical Chemistry (WAC) model, incorporating environmental, economic, and efficiency aspects; and **Sustainability** quantified alignment with UN SDGs, including energy minimization, waste valorization, and safety. Each parameter was normalized on a 0–1 scale, and the composite score calculated as N + Q + S. Benchmarking employed Koel’s Pyramid software, which visualizes comparative performance across dimensions, enabling transparent evaluation and reproducibility. The novelty of this work lies in the development of a multifunctional HA–nCaO composite that delivers substantially higher remediation performance (COD removal 82.4%, TOC 76.1%, color 89.5% within 90 min at 5 g/L) while simultaneously neutralizing vinasse pH to 7.6 ± 0.2 without external alkali addition. Unlike ZnO-based photocatalysis with limited COD reduction, lignin-mediated FeNPs that focus on biohydrogen rather than remediation, or NiNPs–RGO systems restricted to analytical sensing, the proposed composite integrates high efficiency, reusability (≥ 68% after four cycles), and computational sustainability metrics (NQS, Koel’s Pyramid) into a single platform. This combination of rapid treatment, intrinsic pH control, regeneration, and quantitative sustainability evaluation represents a unique advancement over existing methods and underscores the contribution of this study to the state of the art. This study, while providing valuable insights into the performance of the HA–nCaO composite, has several limitations. Kinetic and isotherm analyses offered an initial understanding of adsorption mechanisms, but additional in situ characterization and detailed investigations of pollutant–adsorbent interactions are necessary to fully elucidate the underlying processes. Furthermore, although the tri-axial NQS index provided a computational evaluation of sustainability, real-world assessments including lifecycle analysis, environmental impact, and cost–benefit evaluations are crucial to confirm the composite’s scalability and eco-efficiency. Addressing these limitations in future studies will strengthen the framework for applying HA–nCaO in sustainable wastewater treatment systems.

## Conclusion

In this study, a sustainable novel hybrid adsorbent composed of nano-calcium oxide functionalized with humic acid (HA–nCaO) was successfully synthesized and applied for the treatment of high-strength sugarcane vinasse obtained from an Egyptian sugar company. This study offers a sustainable and scalable solution for the valorization of CaO and humic substances into a high-performance adsorbent suitable for vinasse treatment. Its dual role in organic load reduction and pH neutralization, combined with its green regeneration potential, makes HA–nCaO a promising material for industrial wastewater treatment, particularly in MENA region sugar-producing sectors. The HA–nCaO composite not only effectively removed pollutants but also self-neutralized vinasse by raising its pH from acidic to near neutral without extra adjustment. Adsorption kinetics followed a pseudo-second-order model, indicating chemisorption, and equilibrium data fit the Langmuir isotherm, showing monolayer adsorption with a high capacity of 416.3 mg/g. The HA–nCaO composite was thoroughly characterized by XRD, FTIR, SEM-EDS, BET, zeta potential, and TGA, confirming its thermally stable, mesoporous structure with enhanced surface area and colloidal stability. Batch experiments showed that under optimized conditions (5 g/L dose, 90 min contact, pH ~ 4.3), the composite achieved high removal efficiencies of 85.3% COD, 76.1% TOC, and 89.5% color from vinasse. Thermodynamic analysis confirmed the process was spontaneous and endothermic, with positive entropy changes reflecting strong interactions and surface restructuring. Additionally, the composite retained over 68% COD removal efficiency after four regeneration cycles using simple water rinsing and low temperature drying, highlighting its reusability, durability, and economic viability. Sustainability evaluation with software computational metrics confirmed with superior results of NQS index scores its alignment with UN Sustainable Development Goals (SDGs).

## Supplementary Information

Below is the link to the electronic supplementary material.

Supplementary Material 1

## Data Availability

The datasets generated and/or analysed during the current study are available from the corresponding author on request.
