# Supplementary material for: Sugarcane vinasse remediation through HA–nCaO within a computational sustainability and green SDG framework
Source: Sci Rep. 2025 Nov 27;15:42542. doi: 10.1038/s41598-025-26216-5 (PMC12663415; doi:10.1038/s41598-025-26216-5)
Supplement: Supplementary file 1 — Supplementary Material 1 [file 41598_2025_26216_MOESM1_ESM.docx]

**"Sugarcane Vinasse Remediation through HA–nCaO within a Computational Sustainability and Green SDG Framework"**

**Mahmoud F. Mubarak^1^, Ahmed M. Saleh ^2,3^**

^1^ Petroleum Applications Department, Egyptian Petroleum Research Institute (EPRI), Ahmed El-Zomer,

Nasr City, Cairo, Egypt.

^2^ Pharmaceutical Chemistry Department, Faculty of Pharmacy, Horus University, Horus 34518, Egypt

^3^ Biosensors Research Lab, Zewail City of Science and Technology, 6th October City, Giza 12578, Egypt

**^*Corresponding authors:^**

**Ahmed M. Saleh**

Pharmaceutical Chemistry Department, Faculty of Pharmacy, Horus University, Horus 34518, Egypt

Biosensors Research Lab, Zewail City of Science and Technology, 6th October City, Giza 12578, Egypt

**^E-mail address:^** [**^asaleh@horus.edu.eg^**](mailto:asaleh@horus.edu.eg)

**Mahmoud F. Mubarak**

Petroleum Applications Department, Egyptian Petroleum Research Institute (EPRI), Ahmed El-Zomer,

Nasr City, Cairo, Egypt.

**^E-mail address:^** [**^fathy8753@yahoo.com^**](mailto:fathy8753@yahoo.com)

**TABLE S1**

Sustainable aspects and coordination relationship with the UN's sustainable development goals.

| SDG Category |  | Application |  | |
| --- | --- | --- | --- | --- |
| GOAL 3: Good Health and Well-being (Social) | Minimizes chronic health risks by removing harmful organics and metals from vinasse-contaminated water. | | |  |
| GOAL 4: Quality Education (Social)  GOAL 5: Gender Equality (Social)  GOAL 6: [Clean Water and Sanitation](https://www.globalgoals.org/goals/6-clean-water-and-sanitation/)  (Environmental)  GOAL 8: Decent Work and Economic Growth (Financial, social and governance)  GOAL 9: Industry, Innovation and Infrastructure (Financial and Governance)  GOAL 10: Reduced Inequalities (Social)  GOAL 11: Sustainable Cities and Communities (Social and environmental)  GOAL 12: Responsible Consumption and Production (Social and environmental)  GOAL 15: Life on Land (Environmental)  GOAL 17: Partnerships to achieve the Goal (Finance, environmental, social) | Promote innovation and provide educational and practical training opportunities.  Reduces the burden on women in researh areas with also improving water security  Achieves efficient vinasse purification—removing COD, TOC, and color—while neutralizing pH without added chemicals.  Creates green job opportunities through waste-to-resource recovery and circular economy pathways.  Demonstrates an innovative, scalable treatment system that advances eco-industrial water technologies.  Affordable and accessible water treatment solutions.  Integrating advanced water purification systems into urban infrastructure promotes environmental health.  Wastewater fostering circular economy principles and sustainable industrial practices  Preventing contamination to protect terrestrial ecosystems and biodiversity.  Promoting global cooperation in research, policy, and technology . | | |  |

**
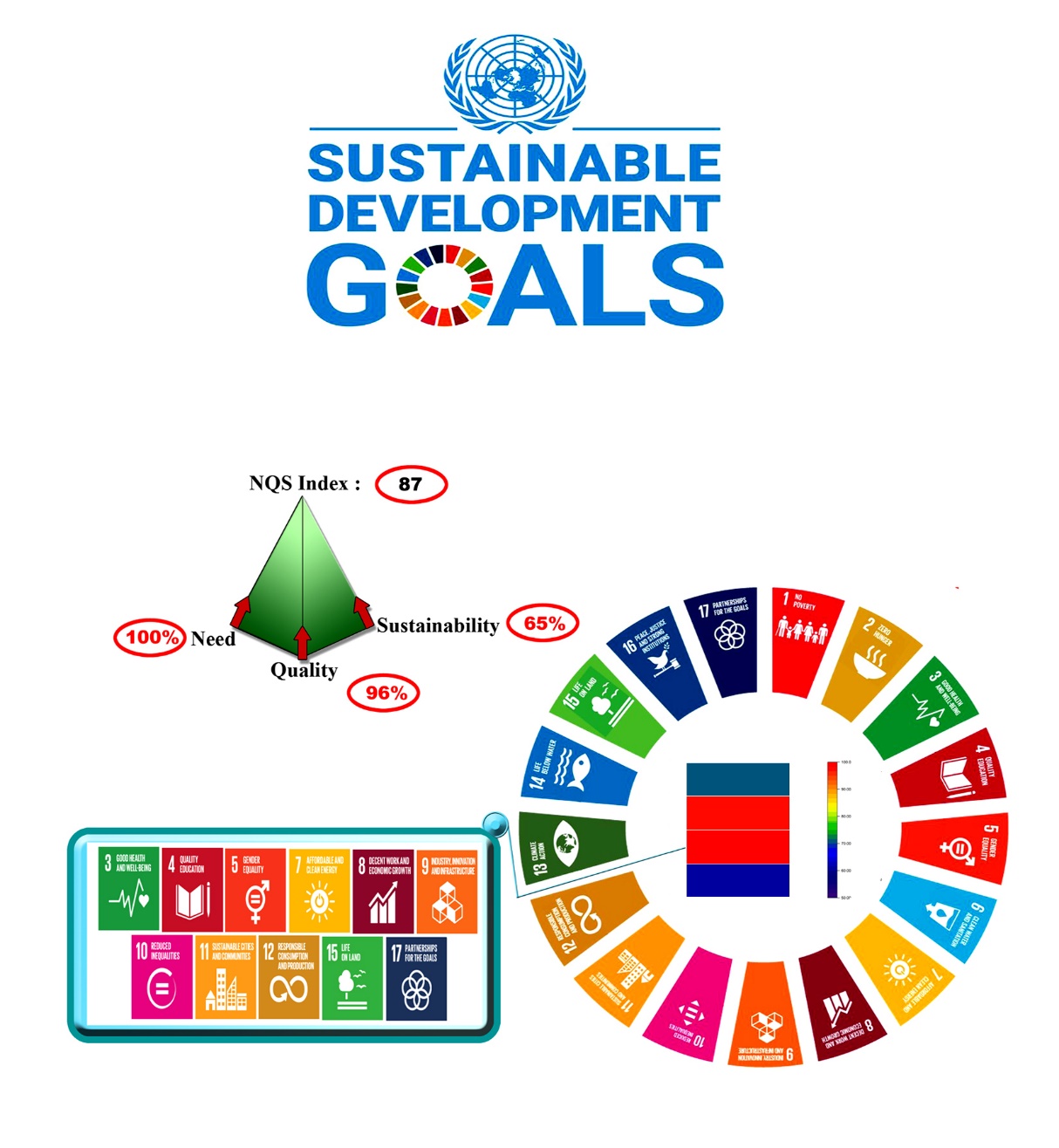
**

**Figure S1:** Sustainability Evaluation using Need Quality Sustainability (NQS) index , Koel’s Pyramid and UN Sustainable Development Goals (SDGs)
